# Impacts of Nano-Composite of Copper and Carbon on Intestinal Luminal Micro-Ecosystem and Mucosal Homeostasis of Yellow-Feather Broilers

**DOI:** 10.3390/microorganisms12112247

**Published:** 2024-11-06

**Authors:** Xianglin Wang, Chunlong Xiao, Shuqing Wu, Qingjie Lin, Shiying Lin, Jing Liu, Dingcheng Ye, Changkang Wang, Pingting Guo

**Affiliations:** 1 College of Animal Science, Fujian Agriculture and Forestry University, Fuzhou 350002, China; 14759106500@163.com (X.W.); 13799729165@163.com (C.X.); 18159801605@163.com (S.W.); lqj18259769095@163.com (Q.L.); lsy15621551956@163.com (S.L.); wangchangkangcn@163.com (C.W.); 2 Institute of Animal Husbandry and Veterinary Medicine, Fujian Academy of Agricultural Sciences, Fuzhou 350013, China; fjliuj@163.com (J.L.); ydcfafu@126.com (D.Y.); 3 Livestock and Poultry Genetic Breeding Key Laboratory of Fujian Province, Fuzhou 350013, China

**Keywords:** Cu-C nano-composites, chicken, mechanical barrier, immunity, bacterial community

## Abstract

The present study was undertaken to evaluate the impacts of nano-composites of copper and carbon (NCCC) on the intestinal luminal micro-ecosystem and mucosal homeostasis of yellow-feather broilers. A total of two-hundred and forty 1-day-old male yellow-feather broilers were randomly allocated into four groups, each with five replications of twelve birds. The control (CON) group received a corn-soybean basal diet, while the N50, N100, and N200 groups were supplemented with 50, 100, and 200 mg/kg of NCCC in basal diets, respectively. The trial duration was 63 days. The findings demonstrated that there were slight impacts of NCCC addition on the intestinal luminal micro-ecosystem of broilers, with the fecal moisture content in the N100 group being slightly higher on Day 3 in the starter phase (*p* < 0.05). The cecal microbiota structure also did not obviously change (*p* > 0.05), in spite of the fall in the relative abundance of the *Ruminococcus torques group* in the N50 group and *norank Clostridia UCG-014* in N200 group (*p* < 0.05). But for intestinal mucosal homeostasis, NCCC played a crucial part in jejunal morphology, tight junction, immunologic status, and antioxidant capacity. There was linear growth in villus height and a quadratic increase in villus height, crypt depth and their ratio with the increase in NCCC dosage (*p* < 0.05), and 100 mg/kg NCCC supplementation could intensify the expression of *CLDN*-3 genes (*p* < 0.05). In addition, IL-4 and IL-10 linearly increased after NCCC treatment (*p* < 0.05), along with some irregular changes in sIgA (*p* < 0.05). In addition, higher jejunal mucosal total antioxidant capacities in N50 and N200 groups were also observed (*p* < 0.05). Overall, NCCC treatment optimized the intestinal mucosa function of broilers in terms of physical barrier and immune and antioxidant capacities, but exerted subtle influence in the luminal environment of yellow-feather broilers. More precisely, dietary supplementation with 50 mg/kg NCCC is recommended for intestinal homeostasis of broilers.

## 1. Introduction

In the past few decades, antibiotics have been utilized as feed additives in poultry production for the purposes of optimizing the intestinal microecosystem and improving growth performance [1]. However, novel functional additives are in urgent need to substitute them after the ban of antibiotics as additives in livestock and poultry production in European and China [2,3].

Copper, an essential trace mineral, plays a critical role in various physiological processes within living organisms and has widespread applications in human life and food production as an antibacterial agent, as well as in animal production [4,5]. In particular, copper exerts growth-promoting effects and other promising benefits in immune functions in poultry production when it is provided at much higher pharmacological levels, i.e., 125 to 250 ppm (in CuSO_4_ form) [6]. Moreover, the price of CuSO_4_ is much lower than other additives, resulting in its wide application in poultry production. However, the absorption interference of other metal ions and environment pollution are also induced when excessive levels of copper are supplied [4]. Consequently, with the aim of averting copper toxicity and environmental contamination while simultaneously retaining its advantageous effects, the exploration of novel high-efficiency copper donors has been underway.

The emerging nanotechnology endows Cu with new physical and chemical features, such as curtailed size and greater active surface area [7]. Cu nanoparticles (Cu-NP) possess higher bioavailability in comparison with traditional Cu providers, and a previous study has shown that supplementing 12 mg Cu-NP per bird in the water during 6 weeks of feeding could promote the immune and antioxidant status of the blood in Ross 308 chickens [8]. Additionally, porous biochar with a high surface area is very suitable for assembling functionalized nanomaterials [9] and is beneficial for growth and reproduction performances of chickens. Better growth performance of broiler chickens was observed after 0.2% inclusion of charcoals from maize cob or Canarium seeds [10]. Additionally, dietary wood (oak) charcoal supplementation significantly increased feed intake, body weight gain and improved feed conversion ratio of broiler chicks during days 8~28, and could significantly reduce number of cracked eggs of laying hens [11].

As a result, a nano-composite of Cu and carbon (NCCC) was selected in our research as a new Cu donor. NCCC is made from natural fibers as the template and Cu^2+^ as the source of copper, and possesses a large quantity of active catalytic sites. Meanwhile, in vitro antibacterial and antifungal effects of NCCC were also observed because the Cu-Cu_2_O-Cu cycle in aerobic and anaerobic environments can destroy microbial redox balance [12]. In view of the above characteristics, we hypothesize that supplementing NCCC in the diet might contribute to the gut micro-ecosystem homeostasis, and immune and redox balance of broilers. Consequently, our study was designed to investigate the influences of dietary NCCC addition on the intestinal homeostasis of broilers to safeguard the commercial application of NCCC-based feed additives for promoting intestinal health in the poultry industry. For this purpose, the extraepithelial environment (the fecal score, moisture content, bacterial load in feces, and the intestinal microbiota structure) and intestinal mucosal function (jejunal morphology, tight junction, immunologic response, and oxidant stress state) of chickens were evaluated in our study.

## 2. Materials and Methods

All management and experimental procedures followed the animal care protocols approved by the Fujian Agriculture and Forestry University Animal Care and Use Ethics Committee (Fuzhou, China, approval ID: PZCASFAFU22002). All experimental protocols were approved and the methods were conducted according to the relevant guidelines and regulations.

### 2.1. Bird Feeding Management

A single-factor completely randomized design was adopted in our study. In total, 240 1-day-old male chickens (32.03 ± 1.35 g) (WENS Foodstuff Group, Yunfu, China) were randomly assigned into four groups: basal diets supplemented with 0 mg/kg NCCC (CON), 50 mg/kg NCCC (N50), 100 mg/kg NCCC (N100), and 200 mg/kg NCCC (N200), each with 5 replicates of 12 broilers. NCCC contains 11.2% copper and 88.8% carbon (Myron Biotechnology, Zhangzhou, China). Copper ions were loaded into biofibers and then fibers were carbonized, forming a copper core and a thin protective carbon shell. The basal diets in the starter, grower and finisher phases of chickens were formulated following the feeding standards of Chinese chickens (NY/T33-2004) and are presented in Supplementary Table S1. The measured contents of copper in 4 groups be found in our recently published paper [12].

The experiment lasted 63 days. Birds were reared in cages (1.2 × 0.8 m) with a rectangular feeder and 4 droplet drinkers. Broilers were exposed to 23 h of light per day. Feed and water were available ad libitum. In the first week, the chicken house temperature was maintained at 35 °C. Subsequently, it was lowered by 2 to 3 °C per week until it reached 22 °C.

### 2.2. Sampling Procedure

Fresh feces in each replicate were collected during the first two weeks of trial to record the fecal score (every day), moisture content (every 3 days) and bacterial load (every 4 days). On D63 of the trial, one bird per replicate was randomly selected to be sacrificed and sampled. The middle of the jejunum was collected and fixed in 10% formalin solution immediately for jejunal morphology detection. The jejunal mucosa was scraped and stored at −80 °C for the determination of immune factors and antioxidant indexes and gene expression of tight junction proteins. Cecal contents were sampled and frozen at −80 °C for 16S rDNA gene amplicon sequencing.

### 2.3. Determination of Fecal Score, Moisture Content and Bacterial Load in Feces

Fecal score (from 1 to 4) was conducted according to the method described by Kong [13]. The scoring standard is as follows: A score of 1 indicated hard, dried and molded feces; a score of 2 indicated molded but moist and loose feces; a score of 3 indicated loose feces with more moisture; and a score of 4 indicated watery, green, or hemorrhage stools.

The detection of moisture content and bacterial load in feces referred to the Chinese National Standards [14,15], respectively.

### 2.4. Bacterial Genomic DNA Extraction and 16S rDNA Gene Amplicon Sequencing

The bacterial genomic DNA of cecal digesta extraction was conducted using the Stool DNA Kit (Omega Bio-tek, Norcross, GA, USA). The bacterial 16S rDNA genes were sequenced as follows. The bacterial V3-V4 regions were amplified by a PCR thermocycler (GeneAmp^®^ 9700, Applied Biosystems, Foster City, CA, USA) using the primers 338F (5′-ACTCCTACGGGAGGCAGCAG-3′) and 806R (5′-GGACTACHVGGGTWTCTAAT-3′). The purified amplicons were mixed in equimolar amounts and then subjected to paired-end sequencing on an Illumina MiSeq PE300 platform in accordance with the standard protocols.

Amplicon sequence variants (ASVs) demarcated by a 99% similarity threshold were assembled using DADA2 (version 1.18). The α-diversity was calculated using Chao, Shannoneven, and Shannon indexes by mothur software (version 1.30.1) [16]. Nonmetric MultiDimensional Scaling (NMDS) at the ASV level, on the basis of unweighted UniFrac distance, was employed to assess the relationships between samples and groups. Additionally, the inter-group difference analysis was conducted via Analysis of Similarities (ANOSIM). Statistically significant differences in the relative abundance of the cecal microbiota were determined using the Kruskal–Wallis test with Tukey’s post hoc test.

All raw sequencing data were deposited into the NCBI Sequence Read Archive database (Accession Number: PRJNA962715).

### 2.5. Intestinal Morphology of Broilers

The intestinal morphology was measured as per Chen’s method [17]. Briefly, after fixed in 10% formalin for 24 h, the jejunum segments were dehydrated with alcohol, washed with xylene, and embedded in paraffin blocks. The intestines were continuously cut into 10 sheets of 5 μm thickness, and then stained with hematoxylin–eosin, and finally sealed with neutral gel. Photographs of the stained sections were taken through the Nikon Eclipse E100 (Nikon Instruments Inc., Tokyo, Japan) and villus height (VH) and crypt depth (CD) were determined via an image analysis system on the Nikon DS-U3 (Nikon Instruments Inc., Tokyo, Japan). VH/CD ratio was also calculated as a vital parameter to evaluate jejunal development.

### 2.6. Detection of Gene Expression Levels by qPCR

Gene expression analysis by qPCR was conducted in accordance with the method described by Tong et al. [18]. The total RNA of jejunal mucosa was extracted using a Total RNA Extraction Kit (Promega Biological Products, Shanghai, China). A spectrophotometer (NanoDrop 2000, Thermo Scientific, Waltham, MA, USA) was utilized to evaluate the RNA concentration and quality. Reverse transcriptase reactions were carried out using a RT Master Mix Kit (Promega Biological Products, Shanghai, China). The cDNA was employed for real-time quantitative PCR (Applied Biosystems 7500, Applied Biosystems, Foster City, CA, USA). The primer sequences are shown in Table 1. The relative gene expression levels were analyzed as per Livak’s method [19] and represented as 2^−ΔΔCT^.

### 2.7. Determination of Jejunal Immune Factors and Antioxidant Indexes

The determination of antioxidant and immunologic indexes was conducted following Tong’s procedure [18]. The jejunal mucosa was isolated and then thoroughly homogenized with cold PBS (weight to volume ratio, 1:9). Thereafter, it was centrifuged at 1000× *g* for 20 min at 4 °C. Subsequently, the concentration of total protein was measured after collecting the supernatant by employing a BCA protein quantitative kit (Beyotime, Shanghai, China). The concentrations of immune factors (Interleukin-2 (IL2), Interleukin-4 (IL4) and Interleukin-10 (IL10)) and antioxidant indexes (the total antioxidant capacity (T-AOC), malondialdehyde (MDA), glutathione (GSH), and oxidized glutathione (GSSG)) were detected using available assay kits (Enzymatic Biotechnology, Shanghai, China). The concentration of secretory Immunoglobulin A (sIgA) was measured by an Enzyme-Linked Immunosorbent Assay (Kamiya Biomedical Company, Tukwila, WA, USA) according to Levkut’s method [20]. The absorbance was examined by a Microplate Reader (PR3100 TSC, Bio Rad, Hercules, CA, USA). The GSH/GSSG ratio was calculated to assess the redox status of glutathione.

### 2.8. Statistical Analysis

Statistical analysis was performed via IBM SPSS Statistics (version 25.0). The variance homogeneities were assessed by Levene’s test and data normality was verified by the Shapiro–Wilk test. One-way analysis of variance (ANOVA) with Duncan’s post hoc test was employed to examine the inter-group disparities. Linear and quadratic comparisons were implemented to ascertain the dose-effect of NCCC on intestinal mucosal homeostasis. A *p*-value < 0.05 was considered statistically significant. The mathematical–statistical models are as follows:

*Y_ij_* = *µ* + *α_i_* + *e_ij_*,

where *Y_ij_* is the value of the trait; *μ* is the overall mean; *α_i_* is the diet effect and *e_ij_* is the random residual error.

*Y_i_* = *α* + *βX_i_* + *e_ij_*,

where *Y_i_* is the dependent variable; *α* is the intercept of the *y*-axis; *β* is the regression coefficient of *Y* on *X*; *X_i_* is the independent variable and *e_ij_* is the random residual error.

*Y* = *b*_0_ + *b*_1_*X* + *b*_2_*X*^2^ + *e*,

where *Y* is the dependent variable; *b*_0_ is the constant term; *b*_1_ and *b*_2_ are the partial regression coefficients; *X* is the independent variable and *e* is the random residual error.

## 3. Results

### 3.1. Fecal Score, Moisture Content and Bacterial Load in Feces

As presented in Table 2 and Table 3, dietary NCCC supplementation exerted no impacts on fecal score and bacterial load of broilers in the starter phase, in comparison with the CON group (*p* > 0.05). However, the fecal moisture content on Day 3 in the N100 group was significantly higher than that in the CON group (*p* < 0.05), with no alternation in the fecal moisture content after 50 or 200 mg/kg NCCC treatment (*p* > 0.05) (Table 4). Furthermore, there was a linear decrease in the fecal score on D13 with the rise in NCCC dosage (*p* < 0.05).

### 3.2. Cecal Microbiota Composition

**Figure 1:**
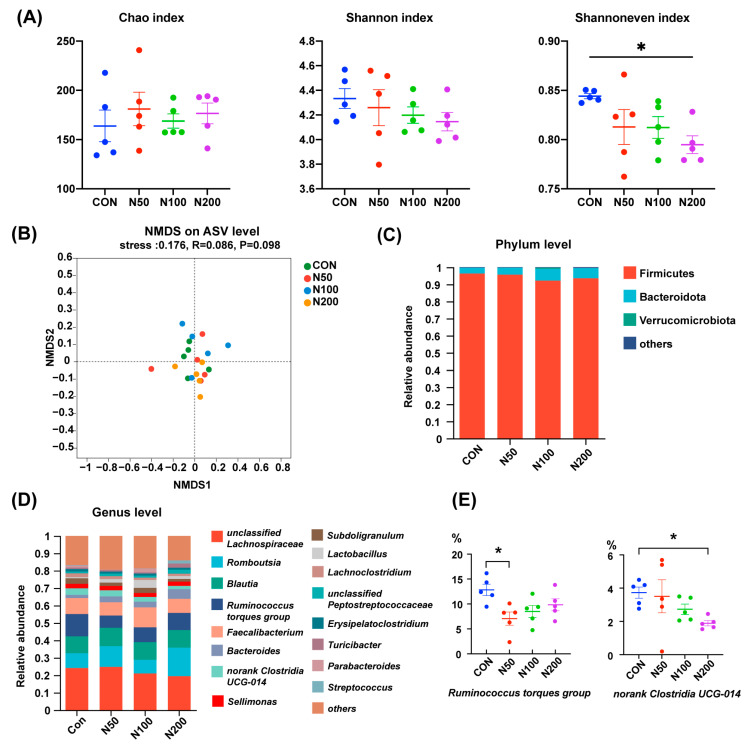
Cecal microbiota alteration after NCCC treatment. (**A**) Chao, Shannon and Shannon even indexes; (**B**) NMDS analysis based on unweighted Unifrac distance and ANOSIM analysis; (**C**) cecal microbiota at phylum level; (**D**) cecal microbiota at genus level; (**E**) two genera with different abundances between groups. Group CON: the basal diet; groups N50, N100 and N200: the basal diets with 50, 100 and 200 mg/kg NCCC, respectively. NMDS: Non-metric multidimensional scaling; ANOSIM: analysis of similarities. * *p* < 0.05.

As displayed in Figure 1A, NCCC addition did not influence the Chao index and Shannon index of cecal microorganisms in birds, while the Shannoneven index in the N200 group was smaller than that of the CON group (*p* < 0.05). NMDS analysis on the basis of an unweighted Unifrac distance showed that the microbiota structure among groups was undifferentiated (Figure 1B).

At the phylum level (Figure 1C), the microbiota in broilers’ cecum were mainly composed of Firmicutes, Bacteroidota and Verrucomicrobiota and the proportions of these three phyla accounted for more than 99%. At the genus level (Figure 1D), *unclassified Lachnospiraceae*, *Romboutsia*, *Blautia*, *Ruminococcus torques group*, *Faecalibacterium* and *Bacteroides* were the dominant genera. The proportions of *Ruminococcus torques group* in the N50 group and *norank Clostridia UCG-014* in the N200 group were less than that of the CON group (*p* < 0.05) (Figure 1E).

### 3.3. Jejunal Morphology

No significant differences in jejunal VH, CD and VH/CD were observed among different groups (Table 5) (*p* > 0.05). However, a linear increase in VH and a quadratic increase in VH, CD and VH/CD were observed by increasing the NCCC dosage (*p* < 0.05).

### 3.4. Gene Expression of Tight Junction Proteins in Jejunum

The impacts of different NCCC concentrations on the gene expression of jejunal tight junction proteins in broilers are shown in Table 6. Compared with CON and N50 groups, 100 mg/kg NCCC supplementation could enhance the expression of the *CLDN*-3 gene (*p* < 0.05). Broilers in the N50 group had a weaker expression of the *CLDN*-1 gene than the N100 and N200 groups, and a linear and quadratic increase in *CLDN*-1 expression with the rise in NCCC supplementation was also observed (*p* < 0.05). However, the gene expression of *OCLN* and *ZO*-1 was not affected by NCCC supplementation.

### 3.5. Immune Factors and Antioxidant Indexes in Jejunal Mucosa

As shown in Table 7, compared with the CON and N50 groups, the jejunal sIgA content was lower in the N100 group and higher in the N200 group. In addition, there was a quadratic relationship between sIgA content and NCCC dosage (*p* < 0.05). No remarkable change in the content of IL-2, IL-4 and IL-10 was found, while there was a linear increase in IL-4 and IL-10 and a quadratic increase in IL-4 by increasing the NCCC addition (*p* < 0.05).

The impact of NCCC addition on jejunal mucosal antioxidant indexes in broilers is also demonstrated in Table 7. The T-AOC values in N50 and N200 groups were higher than the CON group, with a linear and quadratic increase by increasing the NCCC dosage (*p* < 0.05). However, the MDA content and GSH/GSSG were not affected by NCCC treatment.

## 4. Discussion

### 4.1. Intestinal Luminal Micro-Ecosystem

Previous research has indicated that chelated copper and nano-copper can minimize Cu excretion and enhance the efficiency of Cu utilization [21,22]. In our study, NCCC was selected as an alternative Cu source and its impacts on the intestinal health of broilers were explored because of its expansive surface area and strong antibacterial properties. We firstly assessed the luminal microecological environment of broilers, while NCCC exerted limited influence herein, with the fecal moisture content in the N100 group slightly higher on a Day 3 in the starter phase and lower cecal proportions of *Ruminococcus torques group* in the N50 group and *norank Clostridia UCG-014* in the N200 group. *Ruminococcus torques* can restrain the growth of *Akkermansia muciniphila* [23]. Meanwhile, extracellular α- and β-glycosidases were secreted by *Ruminococcus torques* to degrade porcine mucin, thereby undermining gut barrier integrity [24]. Recent research showed that *Ruminococcus torques* was capable of promoting white adipose tissue thermogenesis by improving the production of taurine-conjugated cholic acid and deoxycholic acid, thus resulting in weight loss [25]. As for Clostridia species, most of them utilize indigestible polysaccharides to generate short-chain fatty acids [26]. Intestinal health is benefited in many ways by their extracellular component and metabolites, including energizing intestinal epithelial cells, enhancing the physical barrier, facilitating the accumulation of mucosal Treg cell and inhibiting NF-κB activation [27,28,29,30]. Accordingly, our results suggested that 50 mg/kg NCCC could act as a light enhancer in intestinal extraepithelial homeostasis, but 200 mg/kg NCCC potentially destabilized the luminal environment.

In contrast to our results, Cu-loaded montmorillonite was reported to heighten the proportions of *Bacteroides* and *Faecalibacterium* in the cecum of chickens, while Cu-loaded chitosan nanoparticles could decrease the amount of *Escherichia coli* in duodenum, jejunum, and cecum, and elevate the number of *Lactobacillus* and *Bifidobacterium* in the cecum of piglets [31,32]. It is widely acknowledged that there is an interaction between Cu and the intestinal luminal micro-ecosystem. Bacterial survival and adaption are altered by Cu through regulating its capacity to combat oxidative stress and generate energy via related metabolic pathways and Cu-binding enzymes [33]. Meanwhile, several mechanisms are employed by microorganisms for the transmembrane transport of copper, Cu transaction between organelles, Cu sequestration by metallothioneins, and copper ion oxidization [34,35]. In addition, a recent study uncovered that luminal mucin is a surprising chaperone of multivalent Cu [36]. Herein, to some extent, the weak impact of NCCC on the intestinal luminal environment in our study is probably due to its small size and high biocompatibility. The higher biocompatibility of NCCC compared to copper sulfate means more Cu absorption into epithelial cells and less Cu detainment in the intestinal lumen, implying that NCCC potentially has a greater impact on mucosa homeostasis. Therefore, we continued our investigation into jejunal mucosal function by examining the jejunal morphology, tight junction, immune function, and antioxidant capacity of chickens.

### 4.2. Intestinal Mucosal Homeostasis

The villus, crypt, and tight junction are the primary components of the intestinal mucosa and serve as the physical barrier against xenobiotic and pathogen invasion. Villus height and crypt depth are also two crucial indexes of the development status of intestinal epithelia. Tight junction proteins, such as Claudins, Occludin, ZO-1, ZO-2, and ZO-3 [18,37], are essential components of the paracellular pathway, which is accountable for the passive passage of water, electrolytes, and other small molecules [38]. In our study, 100 mg/kg NCCC addition strengthened the jejunal expression of *CLDN*-3, and jejunal villus height was improved with the rise in NCCC dosage (linear growth), indicating that NCCC could reinforce the physical barrier of broilers. Meanwhile, several novel Cu carriers, like Cu^2+^-loaded montmorillonite and copper-loaded chitosan nanoparticles, were also reported to reverse the intestinal barrier dysfunction induced by excess traditional Cu (in CuSO_4_ form) [31,39,40].

Cu is a component of the active sites of many metalloenzymes, for example, cytochrome oxidase, superoxide dismutase, lysyl oxidase, and tyrosinase, and is involved in all sorts of physiological processes, like cellular respiration, energy production, and antioxidant and immunologic regulation [41]. Hence, we further measured several representative immune factors and antioxidant indicators in jejunal mucosa.

Our results suggested that 50 mg/kg NCCC supplementation improved immune function by elevating the contents of sIgA. SIgA maintains a balanced immune response. It not only safeguards against harmful microbes but also prevents reactions against beneficial bacteria and environmental proteins (such as food antigens) [42]. In our previous study, serum IL-10 levels were raised by 200 mg/kg NCCC treatment, while serum IL-4 levels increased both linearly and quadratically when the NCCC dosage increased [12], which is in line with our present results in jejunal mucosa. Similarly, the supplementation of Cu-loaded montmorillonite has been reported to lower the gene expression levels of jejunal *IL-1β*, *IL-6*, and *TNF-α*, raise gene expression levels of jejunal *IL-4* and *IL-10*, and boost the serum immunoglobulins levels in chickens [32].

Additionally, improved antioxidant capacity was noted in chickens and pigs with Cu-NP, NP, CuO-NP, or Cu-loaded montmorillonite supplementation [32,43,44,45]. Consistent with the above results, the higher jejunal T-AOC in N50 and N200 groups in our study indicated that the total non-enzymatic antioxidant capacity in broiler jejunum was improved by 50 or 200 mg/kg NCCC supplementation. Copper takes on various roles in biological electron and oxygen transportation, which is in favor of the interconversion between cuprous (Cu(I)) and cupric (Cu(II)) states [46]. The antioxidant functions of glutathione and CuZn-SOD, which are integral components of the cellular antioxidant defense system, are Cu-dependent [5]. Moreover, Cu deficiency would attenuate the activity of catalase [47]. To our regret, the activities of the jejunal antioxidant enzyme were not measured due to the damage of samples during the COVID-19 pandemic, resulting in the lack of evidence about the impacts of Cu on enzymatic antioxidant capacity.

## 5. Conclusions

In conclusion, nano-composites of copper and carbon treatment can improve intestinal mucosa health to a certain degree, despite only the slight effects reported on the luminal micro-ecosystem of yellow-feather broilers. Considering the experimental results, environmental hazards, and material cost, the optimal dosage of nano-composites of copper and carbon is suggested to be 50 mg/kg.

## Figures and Tables

**Table 1:** Primer information of housekeeping gene and target genes.

Genes	Primer Sequences (5′→3′)	Accession Number	Annealing Temperature	Product Size/bp
GAPDH	F: 5′-GGTGGCCATCAATGATCCCT-3′	NM_204305.2	56	174
	R: 5′-GCCCATTTGATGTTGCTGGG-3′			
OCLN	F: 5′-AGTTCGACACCGACCTGAAG-3′	XM_025144247.2	55	124
	R: 5′-TCCTGGTATTGAGGGCTGTC-3′			
CLDN-1	F: 5′-TCCTGGGTCTGGTTGGTGTGTT-3′	NM_001013611.2	59	172
	R: 5′-CGAGCCACTCTGTTGCCATACC-3′			
CLDN-3	F: 5′-CCTTCATCGGCAACAACATCGT-3′	NM_204202.2	58	114
	R: 5′-CCAGCATGGAGTCGTACACCTT-3′			
ZO-1	F: 5′-GGTGCTTCCAGTGCCAACAGAA-3′	XM_046899248.1.1	57	186
	R: 5′-GCTTGCCAACCGTAGACCATACTC-3′			

**Table 2:** Impacts of NCCC addition on fecal score of yellow-feather broilers.

Days of Age	Groups				SEM	*p*-Value		
	CON	N50	N100	N200		ANOVA	Linear	Quadratic
D1	3.40	3.20	3.30	3.40	0.055	0.547	0.844	0.404
D2	3.30	3.30	3.30	3.40	0.055	0.907	0.554	0.767
D3	2.30	2.30	2.30	2.20	0.057	0.917	0.571	0.784
D4	2.50	2.20	2.30	2.40	0.073	0.543	0.770	0.399
D5	2.30	2.00	2.10	2.10	0.050	0.186	0.272	0.173
D6	2.00	1.90	2.00	1.90	0.050	0.828	0.667	0.914
D7	1.80	1.80	1.70	1.90	0.050	0.698	0.702	0.647
D8	1.60	1.30	1.40	1.30	0.086	0.600	0.310	0.515
D9	1.80	1.40	1.80	1.50	0.088	0.258	0.539	0.803
D10	1.50	1.10	1.20	1.40	0.091	0.424	0.815	0.267
D11	1.70	1.50	1.50	1.20	0.099	0.379	0.091	0.242
D12	1.40	1.00	1.10	1.10	0.064	0.130	0.167	0.106
D13	1.40	1.30	1.10	1.00	0.067	0.125	0.015	0.056
D14	1.10	1.10	1.00	1.00	0.034	0.585	0.202	0.453

Note: group CON: the basal diet; groups N50, N100 and N200: the basal diets with 50, 100 and 200 mg/kg NCCC, respectively. The scoring standard is as follows: A score of 1 indicated hard, dried and molded feces; a score of 2 indicated molded but moist and loose feces; a score of 3 indicated loose feces with more moisture; and a score of 4 indicated watery, green, or hemorrhage stools.

**Table 3:** Influences of NCCC addition on bacterial load in feces of yellow-feather broilers at different days of age (lg(CFU/g)).

Days of Age	Groups				SEM	*p*-Value		
	CON	N50	N100	N200		Diet	Linear	Quadratic
D4	5.48	5.90	5.25	5.73	0.346	0.941	0.852	0.532
D8	7.31	7.02	7.20	6.75	0.237	0.871	0.495	0.786
D12	7.47	7.78	7.66	8.34	0.271	0.757	0.877	0.937

Note: *n* = 5.

**Table 4:** Effects of NCCC on fecal moisture content in yellow-feather broilers at different days of age (%).

Days of Age	Groups				SEM	*p*-Value		
	CON	N50	N100	N200		Diet	Linear	Quadratic
D3	77.90 ^b^	77.29 ^b^	79.65 ^a^	78.03 ^b^	0.290	0.013	0.298	0.407
D5	77.53	76.64	77.98	77.36	0.385	0.701	0.822	0.962
D7	77.70	76.68	78.01	76.93	0.337	0.489	0.748	0.950
D9	78.02	77.18	78.03	77.53	0.244	0.573	0.785	0.913
D11	78.19	77.91	78.81	77.97	0.211	0.445	0.919	0.815
D13	75.83	75.44	77.17	76.32	0.327	0.287	0.287	0.544

Note: *n* = 5. ^a,b^ Means within the same row that lack the same superscript show significant differences (*p* < 0.05).

**Table 5:** Impacts of NCCC addition on jejunal development of yellow-feather broilers.

Items	Groups				SEM	*p*-Value		
	CON	N50	N100	N200		Diet	Linear	Quadratic
VH (μm)	866.4	1091	1153	1419	121.94	0.078	0.007	<0.001
CD (μm)	187.2	176.5	185.5	220.5	15.58	0.333	0.124	<0.001
VH/CD	4.67	6.32	6.56	6.95	0.775	0.274	0.081	<0.001

Note: *n* = 5. VH: villi height; CD: crypts depth; VH/CD: the ratio of villus height to crypt depth.

**Table 6:** Effects of NCCC supplementation on gene expression of jejunal tight junction proteins in yellow-feather broilers.

Genes	Groups				SEM	*p*-Value		
	CON	N50	N100	N200		Diet	Linear	Quadratic
*OCLN*	1.00	1.01	0.72	0.85	0.051	0.132	0.103	0.234
*CLDN*-1	1.00 ^ab^	0.64 ^b^	1.23 ^a^	1.33 ^a^	0.079	0.002	0.021	0.018
*CLDN*-3	1.00 ^b^	0.85 ^b^	1.79 ^a^	1.26 ^ab^	0.107	0.003	0.075	0.140
*ZO*-1	1.00	1.34	1.10	1.14	0.055	0.138	0.850	0.380

Note: *n* = 5. ^a,b^ Means within the same row that lack the same superscript show significant differences (*p* < 0.05). *OCLN*: Occludin; *CLDN*-1: Claudin 1; *CLDN*-3: Claudin 3; *ZO*-1: Zonula occludens protein 1.

**Table 7:** Influences of NCCC addition on jejunal mucosal immune factors and antioxidant indexes of yellow-feather broilers.

Items	Groups				SEM	*p*-Value		
	CON	N50	N100	N200		Diet	Linear	Quadratic
**Immune factors**								
sIgA (ng/mg prot)	1596.01 ^b^	1549.98 ^b^	1413.30 ^c^	1830.26 ^a^	45.24	<0.001	0.110	0.002
IL-2 (ng/mg prot)	259.07	287.22	296.01	285.58	8.930	0.529	0.280	0.319
IL-4 (pg/mg prot)	69.08	71.14	82.53	83.29	2.407	0.054	0.009	0.036
IL-10 (pg/mg prot)	38.86	39.09	41.58	46.78	1.419	0.166	0.035	0.073
**Antioxidant indexes**								
MDA (nmol/mg prot)	9.68	10.41	8.81	10.62	0.291	0.175	0.504	0.700
T-AOC (U/mg prot)	23.96 ^b^	37.59 ^a^	32.50 ^ab^	38.57 ^a^	2.036	0.016	0.015	0.032
GSH/GSSG	6.05	5.89	6.17	5.98	0.062	0.485	0.909	0.988

Note: *n* = 5. ^a–c^ Means within the same row that lack the same superscript show significant differences (*p* < 0.05). sIgA: secretory immunoglobulin A; IL-2: Interleukin 2; IL-4: Interleukin 4; IL-10: Interleukin 10. MDA: malondialdehyde; T-AOC: total antioxidant capacity; CSH/GSSG: the ratio of reduced glutathione to oxidized glutathione.

## Data Availability

The data presented in this study are available within the article.

## Supplementary Materials

The following supporting information can be downloaded at https://www.mdpi.com/article/10.3390/microorganisms12112247/s1: Supplementary Table S1: The ingredient composition and nutrient levels of diets (air-dry basis, %).
