# Supplementary material for: Impacts of Nano-Composite of Copper and Carbon on Intestinal Luminal Micro-Ecosystem and Mucosal Homeostasis of Yellow-Feather Broilers
Source: Microorganisms. 2024 Nov 6;12(11):2247. doi: 10.3390/microorganisms12112247 (PMC11596944; doi:10.3390/microorganisms12112247)
Supplement: Supplementary file 1 [file microorganisms-12-02247-s001.zip › microorganisms-3256080-supplementary.pdf]

Supplementary Table S1 The ingredient composition and nutrient levels of diets (air-dry basis, %).

Items	Starter phase (d 1 to d 21)	Grower phase (d 22 to d 42)	Finisher phase (d 43 to d 63)
**Ingredients**			
Corn	57.00	64.00	72.00
Soybean meal	32.00	26.40	20.00
Expanded soybean	6.60	6.00	4.50
Limestone powder	1.30	1.00	1.00
Dicalcium phosphate	1.60	1.20	1.10
DL-Met	0.20	0.10	0.10
NaCl	0.30	0.30	0.30
1) Premix	1.00	1.00	1.00
Total	100.0	100.00	100.00
**2) Nutrient levels**			
Metabolizable energy (MJ/kg)	11.95	12.36	12.89
Crude protein (%)	21.00	18.90	15.98
Calcium (%)	0.80	0.77	0.60
Total phosphorus (%)	0.55	0.54	0.50
Available phosphorus (%)	0.31	0.33	0.30
Lysine (%)	1.10	0.96	0.75
Met+Cys (%)	0.97	0.93	0.67

Note: 1) Premix supplied per kg diet in the Starter phase: vitamin A 5 000 IU, vitamin B_1 1.3 mg, vitamin B_2 3.6 mg, vitamin B_6 2.5 mg, vitamin B_{12} 0.01 mg, vitamin D_3 1 000 IU, vitamin E 10 IU, vitamin K_3 0.5 mg, biotin 0.15 mg, folic acid 0.55 mg, pantothenic acid 10 mg, nicotinic acid 35 mg, choline choride 1 000 mg, Cu (as copper sulfate) 8 mg, Fe (as ferrous sulfate) 80 mg, Mn (as manganese sulfate) 80 mg, Zn (as zinc sulfate) 60 mg, I (as potassium iodide) 0.35 mg, and Se (as sodium selenite) 0.15 mg.

Premix supplied per kg diet in the Grower phase: vitamin A 5 000 IU, vitamin B_1 1.3 mg, vitamin B_2 3.6 mg, vitamin B_6 2.5 mg, vitamin B_{12} 0.01 mg, vitamin D_3 1 000 IU, vitamin E 10 IU, vitamin K_3 0.5 mg, biotin 0.15 mg, folic acid 0.55 mg, pantothenic

acid 10 mg, nicotinic acid 30 mg, choline chloride 750 mg, Cu (as copper sulfate) 8 mg, Fe (as ferrous sulfate) 80 mg, Mn (as manganese sulfate) 80 mg, Zn (as zinc sulfate) 60 mg, I (as potassium iodide) 0.35 mg, and Se (as sodium selenite) 0.15 mg.

Premix supplied per kg diet in the Finisher phase: vitamin A 5 000 IU, vitamin B_1 1.3 mg, vitamin B_2 3.0 mg, vitamin B_6 2.5 mg, vitamin B_{12} 0.01 mg, vitamin D_3 1 000 IU, vitamin E 10 IU, vitamin K_3 0.5 mg, biotin 0.15 mg, folic acid 0.55 mg, pantothenic acid 10 mg, nicotinic acid 25 mg, choline chloride 500 mg, Cu (as copper sulfate) 8 mg, Fe (as ferrous sulfate) 80 mg, Mn (as manganese sulfate) 80 mg, Zn (as zinc sulfate) 60 mg, I (as potassium iodide) 0.35 mg, and Se (as sodium selenite) 0.15 mg.

2) Nutrient levels are all calculated values.
